# Erasing Sensorimotor Memories via PKMζ Inhibition

**DOI:** 10.1371/journal.pone.0011125

**Published:** 2010-06-15

**Authors:** Lee Michael von Kraus, Todd Charlton Sacktor, Joseph Thachil Francis

**Affiliations:** 1 Graduate Program in Neural and Behavioral Science, State University of New York Downstate Medical Center, Brooklyn, New York, United States of America; 2 The Robert Furchgott Center for Neural and Behavioral Science, State University of New York Downstate Medical Center, Brooklyn, New York, United States of America; 3 Departments of Physiology and Pharmacology, State University of New York Downstate Medical Center, Brooklyn, New York, United States of America; 4 Department of Neurology, State University of New York Downstate Medical Center, Brooklyn, New York, United States of America; 5 Graduate Program in Biomedical Engineering, State University of New York Downstate Medical Center, Brooklyn, New York, United States of America; Mount Sinai School of Medicine, United States of America

## Abstract

Sensorimotor cortex has a role in procedural learning. Previous studies suggested that this learning is subserved by long-term potentiation (LTP), which is in turn maintained by the persistently active kinase, protein kinase Mzeta (PKMζ). Whereas the role of PKMζ in animal models of declarative knowledge is established, its effect on procedural knowledge is not well understood. Here we show that PKMζ inhibition, via injection of zeta inhibitory peptide (ZIP) into the rat sensorimotor cortex, disrupts sensorimotor memories for a skilled reaching task even after several weeks of training. The rate of relearning the task after the memory disruption by ZIP was indistinguishable from the rate of initial learning, suggesting no significant savings after the memory loss. These results indicate a shared molecular mechanism of storage for declarative and procedural forms of memory.

## Introduction

Memories have been classified into several varieties characterized by different acquisition paradigms, temporal stability, and neural and molecular substrates. Procedural memories, like the skill of riding a bicycle, are thought to be fundamentally different from declarative (explicit) memories because they accumulate slowly through repetition, are expressed automatically in performance, rather than consciously through explicit knowledge, and are subserved by a separate neural system [1], [2]. However, both forms of long-term memory have been proposed to be mediated by the strengthening of synaptic connections through long-term potentiation (LTP) [3]–[5].

In recent years the persistent activity of an atypical and autonomously active isoform of protein kinase C, PKMζ [6], has been shown necessary for the maintenance of LTP and the storage of spatial memories involving the hippocampus, a region required for declarative memory [4]. It was later shown that PKMζ activity in the hippocampus, gustatory cortex, and basolateral amygdala is necessary for several forms of specific and accurately learned memories, but PKMζ has not yet been proven necessary for procedural memories [5], [7].

We therefore tested whether PKMζ activity is necessary for the maintenance of a well-established, consolidated, skilled sensorimotor memory (a paradigmatic procedural memory). Specifically, we tested whether the PKMζ inhibitor, zeta inhibitory peptide (ZIP), disrupts performance on a rat reach-to-grasp and retrieve task. This task has previously been associated with changes in sensorimotor cortex cutaneous receptive field size, baseline level of synaptic transmission and the ability to induce LTP, and the number and stability of dendritic spines [8]–[13].

## Materials and Methods

### Ethics Statement

All work adhered to NIH guidelines and was approved by SUNY Downstate's IACUC (permit #: 02-409-09).

### Animals

Twenty two adult female Long-Evans rats (375–450 g) were used. Eleven rats were randomly selected to be intracortically injected with ZIP (10 nmol/µl) and 11 with saline. Five ZIP/control pairs were injected in the sensorimotor cortex at posterior: 1.5 mm; lateral: 1.5 mm and 2.5 mm, relative to Bregma, while the remaining six pairs were injected in the motor cortex: anterior 1.5 mm; lateral: 1.5 mm and 2.5 mm, relative to Bregma. Of the animals injected in posterior sensorimotor cortex, ZIP was injected with a 24 hr delay after the last training episode for two rats, and with a 4 hr delay for three rats; the results were indistinguishable and therefore combined.

### Task paradigm

**Figure 1:**
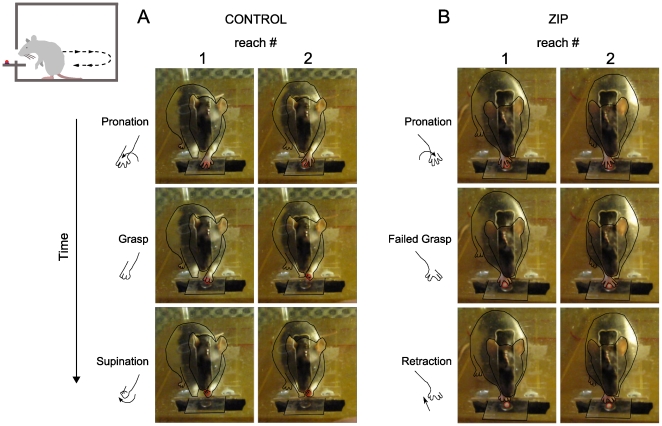
Representative video clips of rats performing reaching behavior after injections of ZIP and saline control into the sensorimotor cortex. Both A) saline and B) ZIP-injected animals first sniff, then pronate their arm in preparation for grasp; the ZIP-injected rat had difficulty in the grasping phase. The rat's body and limbs are outlined to aid viewing relevant features. Insert above left shows illustration of the reaching task, including reach and subsequent stance reset (dashed arrows).

Rats were food deprived to ∼85% of free-feeding body weight and then trained to reach through a narrow vertical slot (15 mm×75 mm) to obtain a food pellet (45 mg ‘dustless precision’ food pellet, Bio-Serve) resting in a metal washer (5 mm ID), 15 mm away on a 40 mm high platform. This same platform extended into the training chamber by 15 mm, towards the rat. For rats injected in the posterior sensorimotor cortex, a 2 mm diameter wooden dowel was placed across the pellet's platform (Fig. 1A, insert) to increase the difficulty of the task. A successful reach, the procedural component of the task, was one in which the rat maintained its grasp and brought the pellet to its mouth. The experimenter did not assist with pellet retrieval. A new pellet was not placed onto the reaching platform by the experimenter until the rat had moved 35 cm to the rear of the cage and then back, resetting its stance. In the case of a successful reach a pellet was placed at the back of the cage as an additional reward. Each rat was trained for 30 min a day until reaching a criterion of >80% average success for 4 consecutive days, with <0.05% SEM; this occurred at least 24 days after the first training session. The experimenter was not informed of the rats' group assignment either prior to or after the ZIP/control injections.

### Surgery

After reaching performance criterion, rats were intracortically injected with ZIP (10 nmol/µl) or saline. Rats were anesthetized with Nembutal and placed in a stereotaxic device. Two small craniotomies were made along with small incisions in the dura to allow for four microinjection sites (two in each hemisphere) with coordinates as described above. For each injection site, a 33 gauge (0.0065” ID, 0.00825” OD) stainless steel cannula was initially lowered to 1.5 mm depth to create a fluid repository before drawing back to administer two injections of 1.25 µl at depths of 1 and 0.5 mm. After the injections, the craniotomies were sealed with bone wax, and the scalp was sutured and treated with antibiotic ointment. Ad libitum food and water were provided over the next 2 days. Measurements for reaching success were begun 4 days after the injections, when previous results have shown ZIP is eliminated [7].

### Drug diffusion analysis

To estimate ZIP spread from our injection sites additional rats were injected with biotinylated ZIP [4], deeply anesthetized after 2.5 hr, perfused with 4% paraformaldehyde, and decapitated. The brain was then removed, vibratome sectioned at 50 µm thickness, and stained with an ABC kit (Vector Laboratories).

**Statistical analysis** Unless otherwise stated, statistics were conducted using ANOVA for within group effects, and ANOVA followed by Tukey's honestly significant difference (HSD) test for comparisons between groups. Linear regression slope and y intercept comparisons were performed using Matlab's ANCOVA function.

## Results

**Figure 2:**
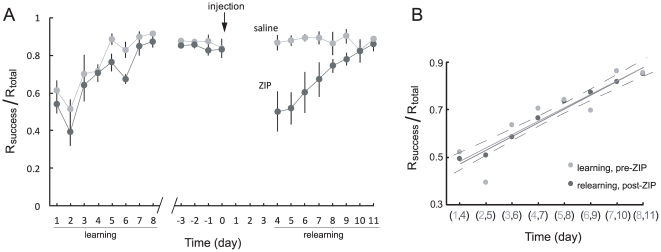
Effects on task performance of ZIP and saline injections into the sensorimotor cortex. A) ZIP, but not saline injection disrupts the retention of sensorimotor memory. Successful reaches/total reaches (R_success_/R_total_) for the initial learning period of 8 days, the 4 days preinjection (days −3 to 0), and, after a delay, the 8 days postinjection (days 4 to 11, with 0 the day of injection). Means ± SEM; 5 animals per group. B) Linear regressions of learning and relearning curves (pre-ZIP, grey solid line; post-ZIP, black solid line). The 95% confidence interval of the pre-ZIP regression (dashed curves) envelopes the post-ZIP regression line.

After 3–4 weeks of training to maximal learned ability on the reaching task (measured as the difference between naïve performance and performance prior to injection), the intracortical injection of ZIP into sensorimotor cortex disrupted the sensorimotor memory (Fig. 1, 2A, Supplementary Fig. S1), causing an 84±21% decrease in learned ability (ANOVA p<0.0001, F = 54). Saline controls showed no change in learned ability post-injection (ANOVA p = 0.98, F = 0.0007). An inactive scrambled version of ZIP [4], [7] had no effect on memory retention (N = 4, ANOVA p = 0.92, comparing performance pre- and post-injection). Comparing all three treatments, only ZIP vs. saline and ZIP vs. scrambled ZIP showed significant differences, whereas saline vs. scrambled ZIP did not (ANOVA followed by Tukey's HSD test; p<0.01, p<0.01, and p = 0.46, respectively). The drug did not affect the number of attempted reaches and stance resets (total reaches and resets per day during relearning days 4–11: saline, 75.5±2.3; ZIP, 69.3±6.4; unpaired 2-tailed t-test p = 0.43), and the animals' gait appeared normal on the first day of task administration post-injection. Animals injected with ZIP anteriorly into the hand/wrist motor area exhibited a smaller memory disruption with a 23±10% decrease in performance (two tailed t-test, p<0.05, N = 6); saline controls showed no change (two tailed t-test, p = 0.78, N = 6). Comparison of treatment effect between groups showed a significant difference (two tailed t-test, p<0.05, N = 6).

Fig. 1 shows video captures of representative reaches for the control and ZIP-injected rats. The stereotypical reaching behaviors of pronation, grasp, and supination were accurately carried out by the control rats (Fig. 1A). In contrast, although the ZIP-injected rats were able to pronate their arms in an accurately aimed reach, they were unable to effectively grasp the food pellet (Fig. 1B). Further studies with high speed, multi-angle video, EMG, and neural recording will be necessary to further characterize these differences.

Fig. 2A shows the average percentage of successful reaches for both control and ZIP groups during initial learning and relearning after injection into the sensorimotor cortex. After reaching asymptotic levels of success, the animals were injected with either saline or ZIP. After 4 days rest, the animals were tested on the reaching task. Following the initial decline in performance to naive levels, the ZIP-injected rats relearned the task, and there was no significant difference between the initial learning and the relearning curves of the ZIP-injected rats (Fig. 2B; ANCOVA p = 0.80, slope; p = 0.35, y intercept). This suggests that there were no significant memory savings or damage to the cortex due to the injections, as also indicated from the lack of change in the control animals' performance post-injection.

**Figure 3:**
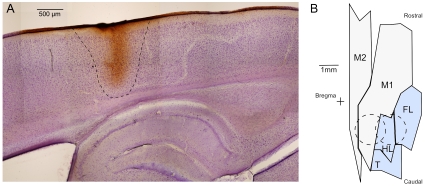
Spread of biotinylated ZIP injections in the sensorimotor cortex. A) Histology showing the extent of diffusion of biotinylated ZIP. Average maximal diffusion of 4 injections is shown by dashed line. There is no diffusion of the biotinylated ZIP to the hippocampus or subcortical structures. Counterstain is cresyl violet. B) Illustration of the diffusion within a single hemisphere from sensorimotor injections sites (dashed circles), mapped onto motor (grey) and sensory (blue) areas: M1, primary motor; M2, secondary motor; and primary sensory (S1) regions, including FL, forelimb; HL, hindlimb; T, trunk; based on [14], [15].

Histological analysis of brain sections indicates the spread of ZIP did not extend into subcortical regions (Fig. 3A), but encompasses several areas involved in skilled reaching including M1, M2, and S1 limb regions [14], [15] (Fig. 3B).

## Discussion

Here we demonstrated that a sensorimotor, procedural memory is dependent on the persistent activity of the autonomously active protein kinase Mζ in the sensorimotor cortex. Previous results have shown that PKMζ is present in neocortex, including motor cortex [16]–[18]. We found that bilateral injections of the PKMζ inhibitor ZIP into the sensorimotor cortex of rats erased the sensorimotor memories learned in a skilled reach-to-grasp task [19]. This is the first demonstration that a procedural memory shares the same underlying molecular mechanism for persistence as declarative memories [4], [5], [7]. Our results support previous studies that have suggested the importance of LTP in sensorimotor memories in the sensorimotor cortices [3], [10] and confirm that the information mediating the reaching task is stored in these brain regions.

There were no apparent postinjection savings in the ZIP-injected rats as indicated by their learning and relearning curves, which were indistinguishable in both slope and y-intercept (Fig. 2B). As our injections did not penetrate subcortically (Fig. 3A), erasing information in the sensorimotor cortex is apparently sufficient to require complete relearning of the task. This suggests that the sensorimotor memory was either maintained by PKMζ-dependent activity entirely within the injected cortical regions, or was distributed over several brain areas with maintenance/retrieval entirely dependent upon PKMζ in the cortex. The latter would be consistent with previous work that proposes a striato-cortico circuit is responsible for habitual, olfaction-guided reaching movements [20], [21].

There are other possible interpretations of our data. For instance, there could have been differences in the level of attention to the task between the ZIP and saline-injected animals. However, if this were the case, we would expect a difference between the number of reaching attempts made between the two groups. No significant difference was observed. In addition, we have presented our results as a percentage of success, thus normalizing the data by the number of attempts. Whether the disruption of sensorimotor memory by ZIP extends to even more basic neural plasticity such as that which establishes and maintains the sensorimotor homunculus will require future investigation.

In recent years there has been a great deal of interest in the concept of consolidation as it pertains to reaching movements [22]–[24]. In our experiments, ZIP disrupted memory after the animals had reached asymptotic performance following several weeks of training, but it cannot be excluded that at some point in the future the memory would become independent of the kinase. Indeed, it will also be interesting in further studies to determine the influence that the passage of time since memory activation has on the PKMζ dependence of procedural memories. It is still unclear what holds consolidated long-term memories in their stable form; however, new synaptic growth has been proposed as one possibility. Whether this new synaptic growth depends upon the persistent activity of PKMζ remains to be determined. Alternatively, synaptogenesis associated with sensorimotor training may play a different role, such as allowing sensorimotor cortex to maintain a balance between excitation and inhibition, or to reestablish the potentiation dynamic range of the neural ensemble [25], [26]. In this sense, the learning-correlated synaptic growth observed by previous researchers may be a homeostatic response to memories maintained by PKMζ activity. Synaptic creation and destruction is a dynamic process, and PKMζ stabilizes synapses [27]. Therefore, what appears to be an increase in synaptogenesis after motor learning may not be due solely to an increase in synaptic formation, but to a shift in the equilibrium between synaptic creation and destruction, thus leading to an overall increase in the number of synapses.

Finally, the results from this study may have direct clinical relevance for the treatment of focal dystonia and chronic pain caused by amputation or peripheral nerve injury, because these are believed to be due to aberrant LTP in sensorimotor areas [28], [29]. As we have now shown that ZIP can erase such sensorimotor memories, the application of ZIP to animal models of these disorders should be tested for therapeutic value.

## Supporting Information

Figure S1 The individual performances of the rats presented in Figure 2. All rats show a deficit in performance after ZIP (blue); none of the rats show a deficit after saline (orange). Solid lines indicate rats trained 4–6 hr prior to injection; dashed lines indicate rats not trained for 24 hr prior to injection. Thick lines indicate mean performances.
(1.45 MB TIF)
