# Supplementary figures and images for: Erasing Sensorimotor Memories via PKMζ Inhibition
Source: PLoS One. 2010 Jun 15;5(6):e11125. doi: 10.1371/journal.pone.0011125 (PMC2886075; doi:10.1371/journal.pone.0011125)

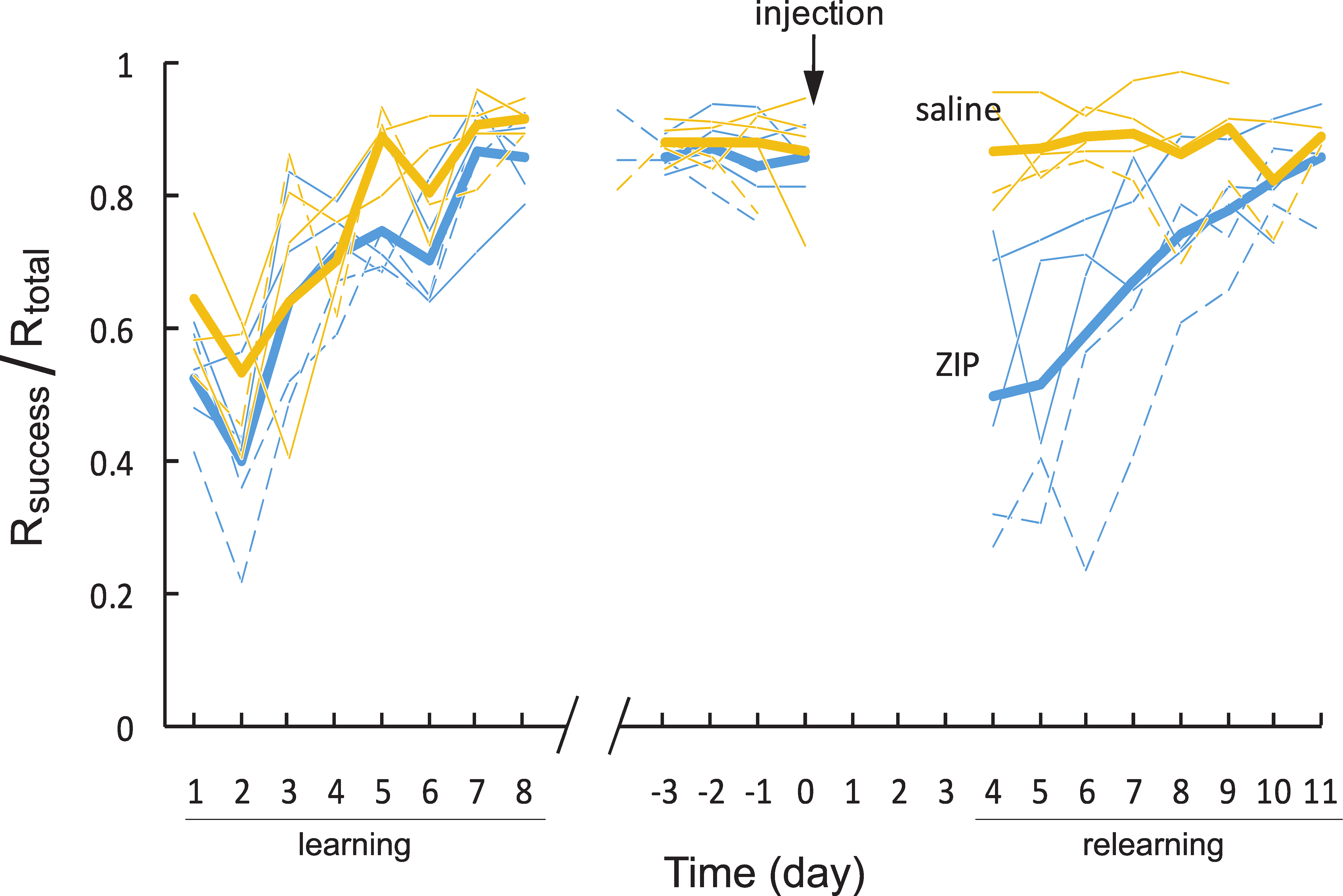

Supplement: Figure S1 — The individual performances of the rats presented in Figure 2. All rats show a deficit in performance after ZIP (blue); none of the rats show a deficit after saline (orange). Solid lines indicate rats trained 4–6 hr prior to injection; dashed lines indicate rats not trained for 24 hr prior to injection. Thick lines indicate mean performances. (1.45 MB TIF) [file pone.0011125.s001.tif]
